# Epidemiology of Digestive Surgical Emergencies in a Sub-Saharan African Country: A Multicentre Pilot Study Protocol

**DOI:** 10.29337/ijsp.186

**Published:** 2023-02-07

**Authors:** Maguatte Faye, Adja Coumba Diallo, Mamadou Ndiaye, Papa Mamadou Faye, Abdourahmane Ndong, Mamadou Cisse

**Affiliations:** 1 General Surgery Department of Dakar Principal Hospital, SN; 2 General Surgery Department of Saint Louis Regional Hospital, US; 3 General Surgery Department of Aristide le Dantec Hospital, SN; 4 General Surgery Department of Dalal Jamm Hospital, SN

**Keywords:** surgery, emergencies, multicenter, mortality, epidemiology

## Abstract

**Introduction:**

Digestive surgical emergencies remains one of the main general surgery activities. Despite the associated mortality rate in low income countries, epidemiologic data about this subject is rare and multicenter studies are even more. We aimed to study an epidemiology of digestive surgical emergencies in Senegal by multicenter protocol.

**Methods and analysis:**

it will a prospective multicenter pilot study from May to July 2022. The patients were from General surgery departments of these teaching hospital in Senegal: Dakar Principal Hospital, Aristide le Dantec Hospital, Dalal Jamm hospital and Saint-Louis Regional Hospital. The Schwartz formula was used. We used a proportion of abdominal surgical emergency of 20%. We had a sample size of 246 patients.

**Ethics and dissemination:**

this research protocol will be submitted to Ethics committee of four hospital that included. The results of this study can help to get better the management of our digestives emergencies and at the same time improve mortality rate.

**Highlights:**

- Digestive surgical emergencies is a public health issue
- Because of high mortality rate in poorest countries, epidemiological data in Senegal must be known
- These information can help to lower associated mortality

## 1. Background

Surgical emergencies remain a public health issue. Indeed, it is estimated that 5 billion people around the world do not have access to surgery when needed particularly in emergency context. This is even more true in low-income countries, where nine out of ten people do not have access to basic surgical care [4]. This constitutes a real burden in terms of morbidity and mortality in underdeveloped countries where there is less than one operating room per 100,000 inhabitant compared to high income countries in Eastern Europe where there is 25 per 100,000 [1]. This is especially important for people requiring emergency surgery, as minutes or hours before treatment has a profound impact on potential disability and chances of survival [5].

These surgical emergencies, whose causes are most often digestive, represent the major part of the activities in general surgery. Indeed, this frequency varies according to the country in Africa about 22.87% in Niger and 20% in Senegal [2,3]. In certain countries like South Africa, an overview of abdominal surgery reveals a high ratio of emergency to elective surgery 58,6% versus 34,5% [13]. The etiologies are diverse, but dominated by acute intestinal obstruction, appendicitis, acute peritonitis and strangulated hernia [1].

These emergencies require multidisciplinary care with close collaboration between surgeons, anesthetist-resuscitators and emergency physicians. Associated mortality is still high in the world’s poorest countries. Despite this high mortality, the epidemiology, patient profile, and their risk factors remain relatively understudied [6]. Several single-centre studies have documented surgical emergencies producing fragmentary data. However, the literature concerning only surgical emergencies, whether traumatic or not, on the scale of a country in Sub-Saharan Africa, remains scarce. This research aims to study the epidemiology of digestive surgical emergencies in Senegal by carrying out a multicenter study.

## 2. Rationale

In Senegal, several studies have been carried out in the field of surgical emergencies. However, these series concerned mostly only non-traumatic acute abdomens and others did not take into account patients who had non operative management [7,8]. In addition, all of these studies were retrospective.

A study by Touré et al. taking into account the place of surgical conditions in medical emergencies showed a prevalence of 20,8% [9]. However, this study included all surgical emergencies whatever the specialty and dates from 20 years ago. Considering the nature and the date of realization of these studies which gave partial information, it would be interesting to carry out a study to update these data.

Our study will have the strength in addition to being multicentric, prospective; to allow us to have a more global view on the acute abdomen operated or treated medically.

The results of this study will be presented in an international conference and published in an international peer reviewed journal.

## 3. Methods

The methodology of this protocol is reported in line with the **STROCSS** guidelines for the reporting of cross-sectional studies in surgery [14]. This protocol has been registered at Research Registry (https://www.researchregistry.com/; Number: 7833).

### 3.1. Objective of the study

This study primary objective is:

- to describe prevalence of each digestive surgical emergencies in Senegal.

The secondary objectives are:

- determine the frequency according to each age class and sex;
- determine the average time of surgical management;
- determine the mortality rate.

### 3.2. Study setting

Senegal is a West African state considered as a low-income country. Its population is about 15 million inhabitants with a pyramidal health system. In Senegal, data from the African Region of the World Health Organization (WHO) show a lack of enough health providers with 0.6 physician per 10 000 populations in 2016 [10] and even fewer of general surgeon compared to USA where there is 9–10 general surgeon per 100,000 inhabitants [1]. Besides, there is an unbalanced repartition of the existent medical doctors and specialists according to National agency of Statistics and demography [11]. The country has 14 medical regions and 76 health districts [12].

The study will be carried out in the General Surgery departments of the following level 1 hospitals:

- Dakar Principal Hospital;
- Aristide le Dantec Hospital (Dakar);
- Dalal Jamm Hospital(Dakar);
- Saint-Louis Regional Hospital.

### 3.3. Type and period of study

It will be a multi-centre cross sectional study in a period of 3 months (May 1st, 2022–30th July 2022). The data collection will be done prospectively.

### 3.4. Inclusion criteria

Patients included will be those with:

- digestive surgical emergency (bowel obstruction, peritonitis, strangulated internal or external hernia, blunt or penetrating trauma);
- traumatic abdominal emergency with parietal or digestive injured;
- whatever the treatment (surgical or non-operative management);
- aged more than15 years old.

### 3.5. Exclusion criteria

Cesareans, gynaecological, urogenital, and vascular emergency surgical procedures will be excluded.

Patients aged lower than 15 years old were excluded to.

### 3.6. Sample size calculation

The formula used to calculate the sample size is Schwartz: N = (Zα^2^.P(1-P))/e^2^.

Zα: = 1.96: reduced difference corresponding to the risk granted (α = 5%, Zα = 1.96).

P: proportion of abdominal surgical emergency = 20%

e: margin of error (set at 5%).

We used a proportion of abdominal surgical emergency of 20% according to 2 previous single center study performed at Aristide le Dantec Hospital in 2002 and 2016 [3,9]. We had a sample size of 246 patients.

### 3.5. Data analysis

The qualitative variables will be described in number with their proportion and the quantitative variables in the form of mean with their standard deviation.

### 3.6. Data collection and entry

Data collection will be prospective on an online survey form on Google form (https://forms.gle/7H5xDfPgJkG7utmv5).

SPSS 26 software will be used for statistical analyses will. Graphs and tables will be made in Excel.

### 3.7. Studied parameters

The studied parameters will be:

- Epidemiological data: hospital, age, gender;
- Diagnostic data: consultation time, symptoms, number of structures consulted before, ASA score, hemoglobin (anemia), hematocrit (hemoconcentration), white blood cell (leukocytosis), creatininemia, imaging modality (ultrasonography, CT scan), diagnosis final;
- Therapeutic data: type of treatment (medical treatment, open surgery, laparoscopic surgery), surgical procedures, death within 30 days.

### 3.8. Ethics and dissemination

Anonymity and confidentiality of information collected in patient will be respected. This research protocol will be submitted to the Ethics Committee of our institution for approval.

The free and informed consent of the patients will be obtained before inclusion. Refusal will in no way prevent it from being accepted for treatment. This research protocol will be submitted to the Ethics Committee of our institution for approval.

## 4. Limitations of study

This study is carrying in 4 teaching hospitals. Inclusion of another hospitals would improve reliability of results about epidemiology of digestives emergencies. We hadn’t funding in our study and this was a barrier for extension of survey to others center.

## Registration of research studies

We have registered our study with unique identifying number: researchregistry7833.

## References

[1] Stewart B, Khanduri P, McCord C, Ohene-Yeboah M, Uranues S, Vega Rivera F, et al. Global disease burden of conditions requiring emergency surgery. Br J Surg. 2014; 101: 9–22. DOI: 10.1002/bjs.9329 24272924

[2] Magagi IA, Adamou H, Habou O, Magagi A, Halidou M, Ganiou K. Urgences chirurgicales digestives en Afrique subsaharienne: étude prospective d’une série de 622 patients à l’Hôpital national de Zinder, NigerDigestive surgical emergencies in Sub-Saharan Africa: a prospective study of a series of 622 patients at the National Hospital of Zinder, Niger. Bull Soc Path Exot. 2017; 110: 191–197. DOI: 10.1007/s13149-016-0499-9 27299912

[3] Ibrahima G, Alassane LP, Mour TM, Ibrahima NP, Boubacar BEH, Bah MD, et al. Prise en charge péri opératoire des urgences chirurgicales abdominales chez l’adulte au CHU Aristide Le Dantec. The Pan Afr Med J. 2016; 24: 190. DOI: 10.11604/pamj.2016.24.190.9929 27795787 PMC5072862

[4] Meara JG, Leather AJM, Hagander L, Alkire BC, Alonso N, Ameh EA, et al. Global Surgery 2030: evidence and solutions for achieving health, welfare, and economic development. Lancet. 2015; 386: 569–624. DOI: 10.1016/S0140-6736(15)60160-X 25924834

[5] Weiser TG, Regenbogen SE, Thompson KD, Haynes AB, Lipsitz SR, Berry WR, et al. An estimation of the global volume of surgery: a modelling strategy based on available data. Lancet. 2008; 372: 139–144. DOI: 10.1016/S0140-6736(08)60878-8 18582931

[6] Havens JM, Peetz AB, Do WS, Cooper Z, Kelly E, Askari R, et al. The excess morbidity and mortality of emergency general surgery. J Trauma Acute Care Surg. 2015; 78: 306–11. DOI: 10.1097/TA.0000000000000517 25757115

[7] Soumah SA, Ba PA, Diallo-Owono FK, Toure CT. Les abdomens aigus chirurgicaux en milieu africain: étude d’une série de 88 cas à l’hô-pital Saint Jean de Dieu de Thiès. Sénégal Surgical acute abdominal emergencies in an African area: study of 88 cases at Saint Jean de Dieu hospital in Thiès. Senegal Bull Med Owendo. 2011; 13(37): 13–16.

[8] Diop PS, Ba PA, Ka I, Ndoye JM, Fall B. Prise en charge diagnostique des abdomens aigus non traumatiques au service des urgences de l’hôpital général de Grand Yoff: à propos de 504 cas. Bull Med Owendo. 2011; 13: 42–46.

[9] Toure CT, Dieng M. Emergency care in tropical areas: status report based on surgical emergencies in Senegal. Medecine Tropicale: Revue Du Corps de Sante Colonial. 2002; 62: 237–241. 12244918

[10] Organization WH. Atlas of African Health Statistics 2016: health situation analysis of the African Region; 2016.

[11] Adjoint DG. Agence Nationale de la Statistique et de la Démographie; 2014.

[12] Ministère de la Santé et de l’Action Sociale (MSAS) Direction Générale de la Santé Plan National de Développement Sanitaire et Social (PNDSS) 2019–2028, Sénégal (2019); n.d.

[13] Vincent UE, Kohler CF, HIV Man D, Tefera A, Lutge E, Clarke DL. The Burden of Emergency Abdominal Surgery Heavily Outweighs Elective Procedures in KwaZulu-Natal Province, South Africa. J Surg Res. 2021; 259: 414–419. DOI: 10.1016/j.jss.2020.09.013 33616075

[14] Mathew G, Agha R, for the STROCSS Group. STROCSS 2021: Strengthening the Reporting of cohort, cross-sectional and case-control studies in Surgery. International Journal of Surgery. 2021; 96: 106165. DOI: 10.1016/j.ijsu.2021.106185 34774726

